# Electricity generation and oxidoreductase potential during dye discoloration by laccase-producing *Ganoderma gibbosum *in fungal fuel cell

**DOI:** 10.1186/s12934-023-02258-0

**Published:** 2023-12-14

**Authors:** Aisha Umar, Islem Abid, Mohammed Antar, Laurent Dufossé, Lobna Hajji-Hedfi, Mohamed S. Elshikh, Abeer El Shahawy, Ahmed M. Abdel-Azeem

**Affiliations:** 1 Institute of Botany, University of the Punjab, Lahore, 54590 Pakistan; 2 Department of Botany and Microbiology, College of Science, King Saud University, 2455, 11451 Riyadh, Saudi Arabia; 3 Department of Plant Science, McGill University, Sainte-Anne-de-Bellevue, Montreal, Quebec, H9X 3V9 Canada; 4 Laboratoire CHEMBIOPRO (Chimie et Biotechnologie des Produits Naturels), Université de La Réunion, ESIROI Département Agroalimentaire, 15 Avenue René Cassin, 97490 Saint-Denis, France; 5 Regional Centre of Agricultural Research of Sidi Bouzid, CRRA, Gafsa Road Km 6, 357, 9100 Sidi Bouzid, Tunisia; 6 Department of Civil Engineering, Faculty of Engineering, Suez Canal University, 41522, Ismailia, Egypt; 7 Botany and Microbiology Department, Faculty of Science, Suez Canal University, 41522, Ismailia, Egypt; 8 Department of Genetics, Faculty of Natural and Agricultural Sciences, University of the Free State, Bloemfontein, 9300 Republic of South Africa

**Keywords:** Green energy, Fungi, Treatment, Energy, Catalyst, Laccase

## Abstract

Color chemicals contaminate pure water constantly discharged from different points and non-point sources. Physical and chemical techniques have certain limitations and complexities for bioenergy production, which motivated the search for a novel sustainable production approaches during dye wastewater treatment. The emerging environmental problem of dye decolorization has attracted scientist's attention to a new, cheap, and economical way to treat dye wastewater and power production via fungal fuel cells. *Ganoderma gibbosum* was fitted in the cathodic region with laccase secretion in the fuel cell. At the same time, dye water was placed in the anodic region to move electrons and produce power. This study treated wastewater using the oxidoreductase enzymes released extracellularly from *Ganoderma gibbosum* for dye Remazol Brilliant Blue R (RBBR) degradation via fungal-based fuel cell. The maximum power density of 14.18 mW/m^2^ and the maximum current density of 35 mA/m^2^ were shown by the concentration of 5 ppm during maximum laccase activity and decolorization of RBBR. The laccase catalysts have gained considerable attention because of eco-friendly and alternative easy handling approaches to chemical methods. Fungal Fuel Cells (FFCs) are efficiently used in dye treatment and electricity production. This article also highlighted the construction of fungal catalytic cells and the enzymatic performance of fungal species in energy production during dye water treatment.

## Introduction

The rapid industrial and global population growths have polluted water and depleted the resources of fossil fuels to fulfil the excessive demand for energy production. Water quality is deteriorating due, for example, among many others, to the continuous mixing of undesirable dye chemicals [1]. The need for water quality improvement is continuously growing due to agricultural, civilization, and industrial activities. Non-point dye sources contaminate valuable water resources. Dye pollutants are hazardous and toxic as multiple industrial applications directly discharge them into the aquatic environment, which leads to pollution; hence, chemical processes are suitable, but often toxic to remediate and eliminate the recalcitrant dyes. Various physical and chemical techniques are used to treat dye-contaminated water. Traditional methods have certain limitations for bioenergy production, because of large spaces, high capital costs, and complexities linked with the production processes [2]. Sustainable bioenergy sources have been increasing worldwide as an alternative to chemical methods for power generation. Biological degradation involves, for some, using microorganisms (fungi, algae, bacteria, and enzymes) and is better than other biological methods (e.g., plants), which utilize a large land areas, exhibit very high sensitivity toward toxic dyes, and require a long consumption time [3]. The fungus can discolor and completely mineralize the dyes [4]. Many industrial lines utilize the synthetic dyes (textile, dyeing, pharmaceutics, cosmetics, and food industries) day by day [5].

The exploration of novel and efficient approaches attracted the attention of environmentalists to clean and remediate the contaminated water bodies. The fungal potential to generate bioelectricity from dye wastewater reduces the conversion costs [6]. Biotic sources exploit the different species of fungi for bioenergy generation. However, little data is available on using a “fungal-mediated electrochemical system” for energy production. Minimal resources, higher prices of fossil fuels, and increasing global warming issues motivated the scientists to design alternative “renewable” energy sources.

Enzymes are an alternative to minimize “pollution” via “Fungal Fuel Cell (FFC)” [8]. The fungal fuel cell is a device that uses a fungal catalyst to generate electricity by oxidizing the dye-based water [9]. These cells also provide electricity directly through biodegradation of the raw materials via fungal cell action [10]. Fungal species have nine times higher potential to generate energy than conventional methods due to the complex enzymes catalytic mechanistic approach [12]. A few researchers believe that this technology could produce electricity during the elimination of dye components via fungal catalytic action [11].

Wood rotting species rapidly grow and degrade the materials quickly, along with bioenergy production. *Ganoderma,* a white rot fungus, is the most efficient fungal genus in break down of synthetic dyes, because these filamentous fungi produce laccase, which catalyzes and degrades the various dyes [7].

In this work, *G. gibbosum* was used as a “bioremediation” in a technique also called the “Oxidative Biocatalytic” approach. The efficiency of this strategy is maximized by using ABTS (2,2ʹ-azino-bis-(3-ethylbenzothiazoline-6-sulfonic) acid redox mediator system. The fungal approach seems to be the best and most cost-effective method for power production during dye-based water treatment [7].

## Objective

To generate electricity during the treatment of dye-based water (substrate) by redox action of laccase from *G. gibbosum* in an eco-friendly manner.

## Materials and methods

### DNA extraction and phylogeny for species identification

The specimens of *Ganoderma gibbosum* were collected from Changa Manga Forest, District Kasur, Punjab, Pakistan (31.0500°N 73.4072°E, 200 m a.s.l) on *Dalbergia sissoo* Roxb. Ex DC. and modified CTAB procedure was followed to extract total genomic DNA from the dried specimens [13]. Molecular identification of the target species, *G. gibbosum*, was performed by comparing its ITS1—5.8S—ITS2 rDNA region sequence data with data on reference strains deposited in GenBank.The consensus of sequence was generated from both ITS1 and ITS2 in BioEdit vers. 7.2.5. The sequences of *G. gibbosum* were compared with reference ITS sequences of GenBank database at the National Center for Biotechnology Information (NCBI), using the basic local alignment search tool (BLAST). Evolutionary distance matrices based on the neighbor-joining algorithm (with max sequence difference of 0.75) were calculated using Kimura’s two-parameter model. Tree topology was inferred by the neighbor-joining method in the program MEGA10 software, with bootstrap values based on 1000 replications. The phylogenetic tree was constructed to identify *Ganoderma* species, where the *Tomophagus colossus* outgroup supports the tree. The studied specimens were deposited in the Mycology Herbarium, Laboratory of Mycology, Institute of Botany, Academy of Sciences of the Republic of Uzbekistan, Tashkent 100125, Uzbekistan (TASM-ZSH). Basidioma was deposited in the Fungarium of Suez Canal University (https://ccinfo.wdcm.org/collection/by_id/1180), at Botany and Microbiology Department, Faculty of Science, Ismailia 41,522, Egypt, under accession number SCUF 1085.

### Qualitative analysis

The pure mycelium of *G. gibbosum* was inoculated on agar media to evaluate the laccase-producing ability of mycelium. Malt Extract Agar media was prepared in *g*/L by adding Malt Extract 7, Agar 10, MgSO_4_·7H_2_O 0.5, K_2_HPO_4_ 0.5, KH_2_PO_4_ 0.5, ZnSO_4_ 0.005, MnSO_4_ 0.05, Peptone 2.5 and Glucose 15 at pH 5.0 (sterilized in an autoclave for 20 min at 121 ℃). Autoclaved media was amended with 0.02% guaiacol to evaluate the laccase-producing ability of the specimen’s pure mycelium. The replicates (plates) were incubated at 30 ℃ for 7 days to check the laccase-producing ability.

### Solution preparation

#### a. Stock of Dye (1000 ppm) and standard solution

Remazol Brilliant Blue R (RBBR) was purchased from Sigma-Aldrich. RBBR (1 *g*) was taken and dissolved in an appropriate amount of distilled water. This solution was transferred into a volumetric flask and made the volume up to 1000 mL using distilled water. This solution was further used for making the standard solutions. 

#### b. Standard solutions

**Fig. 1:**
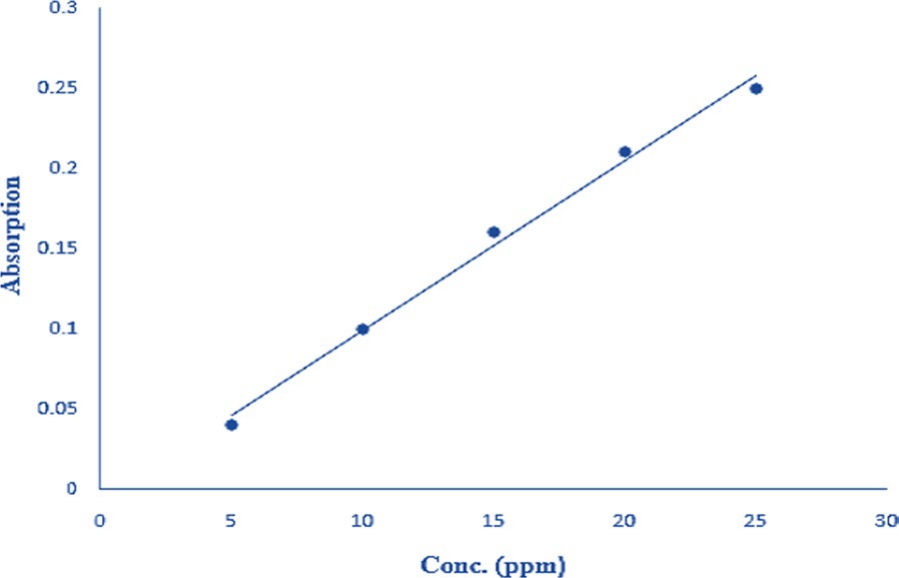
Calibration line for RBBR dye concentration (ppm) against the absorbance

Standard solutions of dye in varying concentrations (5 ppm, 10 ppm, 15 ppm, 20 ppm and 25 ppm) were prepared from the above stock solution. These solutions were prepared by taking 0.5, 1.0, 1.5, 2.0 and 2.5 mL from the stock solution and diluting by adding 100 mL of distilled water. The absorbance was measured by a spectrophotometer at 617 nm, and a calibration line for RBBR was drawn (Fig. 1).

### Fungal fuel cell construction

#### a. Anodic chamber

The anode chamber (1 L) was made from a cylindrical polypropylene bottle. The phosphate buffer solution (0.5 L; pH 6.0) and 100 mL of RBBR solution of varying concentrations (5 ppm, 10 ppm, 15 ppm, 20 ppm and 25 ppm) were used for decolorization (%) under a complete air-tight chamber.

#### b. Cathodic chamber

**Fig. 2:**
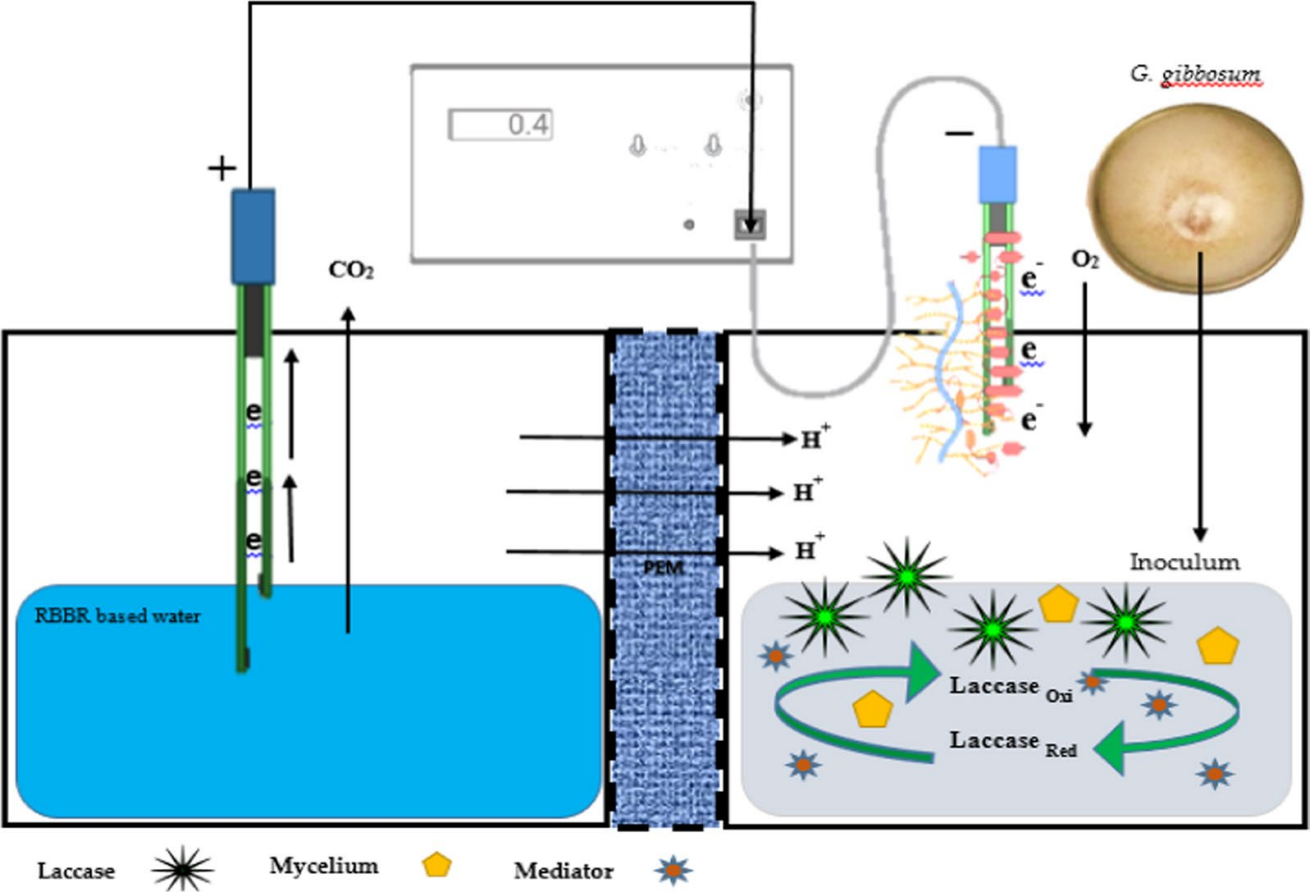
Fungal fuel cell. The cathodic region (right) contains extracellular laccase of fungal mycelium, and the anodic region (left) comprises ABTS-based water

A cathodic chamber called a fungal chamber (0.5 L) was also made from a cylindrical polypropylene bottle. In the cathodic chamber, the pure mycelium of *G. gibbosum* was inoculated in the liquid broth (100 mL). The liquid broth comprised macro and micronutrients in g/mL (yeast extract 5 *g*, starch 1 *g*, while tracers (MgSO_4_⋅7H_2_O 0.5 *g*, NaCl 0.5 *g*, FeSO_4_⋅7H_2_O 0.5 *g*, KH_2_PO_4_ 0.046 *g*, K_2_HPO_4_ 0.1 *g*, CaCl_2_ 0.5 *g*, ZnSO_4_ 0.02 *g*, CuSO_4_⋅5H_2_O 0.5 *g*, H_4_PO_4_ 1.0 *g*, Na_4_HPO_4_ 0.05 *g*, MnSO_4_ 0.001 *g*, ZnSO_4_ 0.001 *g*). The cathodic chamber was continuously stirred and saturated with air. As previously reported, the anodic and cathodic chambers were filled with activated carbon fiber. This FC was operated at 27 ± 2 ℃ at pH 6.5. To assess the contribution of mediators on the biocathode FC, 0.2 mM ABTS was dosed to the catholyte in FCs for the stability of voltage; when the voltage declined to below 50 mV, 0.2 mM ABTS was dosed again. The voltage output in each batch test was measured for 5 days, and readings were noted every 3 h (Fig. 2).

### Electrochemical analyses

A multimeter (‘model 2700 Keithley Instruments, Cleveland’) was used to measure the voltage (V) of the FC and Current (I) was calculated from the external resistance (R) using an equation. Equation 1 was used to plot the polarization curve.

1 $${\text{I = }}{{\text{V}} \mathord{\left/ {\vphantom {{\text{V}} {\text{R}}}} \right. \kern-0pt} {\text{R}}}$$

Power (P) was calculated from the voltage and current equation and divided by the anode surface area to obtain power density. Equation 2 was used to plot the power density curves.

2 $${\text{P = I}}_{{\text{X}}} {\text{V}}$$

The internal resistance of FC was determined using the power density peak and polarization slope method [14]. Constant voltage was best for fuel cell performance and then disconnected (3 h) to maximize the voltage.

### Quantitative analysis of laccase activity

The reaction mixture contained 2.3 mL of 0.1 M sodium acetate buffer (pH 5.0), 0.2 mL of 0.1 mM guaiacol, and a volume of catholyte (0.5 mL) of *Ganoderma* made the total volume of 3 mL. UV Spectrophotometer (Thermo Fisher) monitored the absorbance at 470 nm (3 min), and the activity was expressed in U/L. The laccase activity is measured in “U”. The Unit of activity is defined as 1 mM of guaiacol oxidized per minute by laccase under assay conditions.

$$\frac{{\text{U}}}{{\text{L}}}{ = }\Delta {\text{Abs470}} * \frac{{{\text{Vt}}}}\epsilon{{{\text{*1*Vs}}}}$$

where:

ε = 6,740 M ^−1^ cm ^−1^ extinction coefficient of guaiacol.

Vt = Total vol. of the reaction mixture (mL).

Vs = Vol. of the sample (mL).

l = Length of the cuvette (1 cm).

### Dye decolorization

The 1.5 mL decolorized solution of different concentrations used in anodic chambered was added separately to Eppendorf. These Eppendorf tubes were incubated at 80 ℃, 160 rpm for 1 h. The decolorized solution was removed from the Eppendorf, and again 1.5 mL of fresh dye solution was added to check the stability of laccase action in dye removal. The decolorization by laccase was determined as a relative decrease in absorbance (617 nm) at a maximum wavelength for dye by the following formula [15].

$$\mathbf{D}(\mathbf{\%})=\frac{100\left(\mathbf{C}1-\mathbf{C}2\right)}{\mathbf{C}1}$$

where D (%) is the decolorization of dye, C1 is the OD of the initial dye system, and C2 is the OD of the dye system after incubation with laccase. The absorbance was measured at 617 nm, and decolorization was expressed in percentage.

### Statistical analysis

The collected data from various parameters were analyzed ± standard deviation (SD) less than 5% of triplicate assays. Statistical analysis was performed by using 1-way ANOVA in SPSS18.0 software. To determine the differences among groups, ANOVA was followed by Tukey’s test as a posthoc analysis at p < 0.05.

## Results 

### Molecular phylogenetic tree

**Fig. 3:**
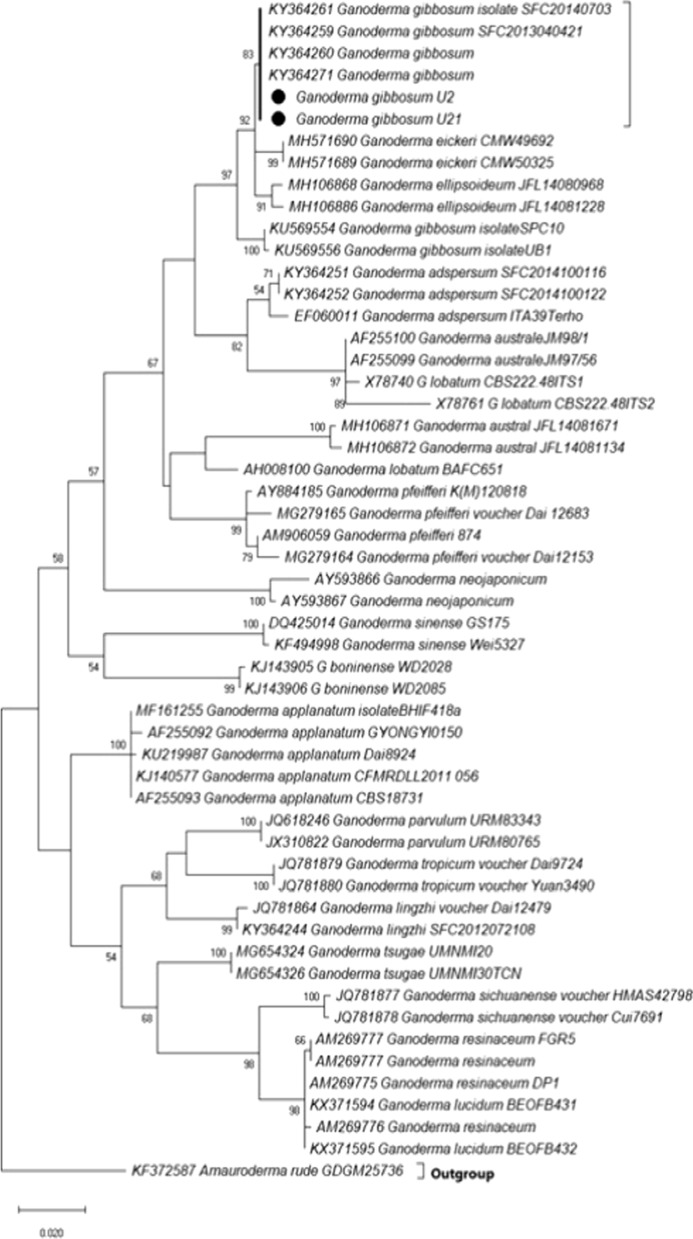
The phylogenetic tree identified *Ganoderma gibbosum* (U2, U21) by ITS marker with 83% bootstrap value

The semi-strict consensus tree was produced by maximum likelihood analysis and informative sites depicted in Fig. 3. The branch that separated *G. gibbosum* from the other *Ganoderma* species in the consensus tree. The 83% bootstrap analysis clustered in well-supported clades forming the monophyletic groups and clustered together in non-laccate species clade (Fig. 3). *G. gibbosum* sequence (U1, U21) used in this study made the clade with *G. gibbosum* sequences collected from literature and GenBank. *Tomophagus colossus* was selected as an outgroup. The sequences generated during this study were deposited in GenBank (OM350446, OM350473). 15 July 2018, Aisha Umar, U21 (GenBank OM350473, TASM-ZSH090); 10 April 2019, Aisha Umar, U1 (GenBank OM350446, TASM-ZSH091).

### Qualitative indication of laccase

**Fig. 4:**
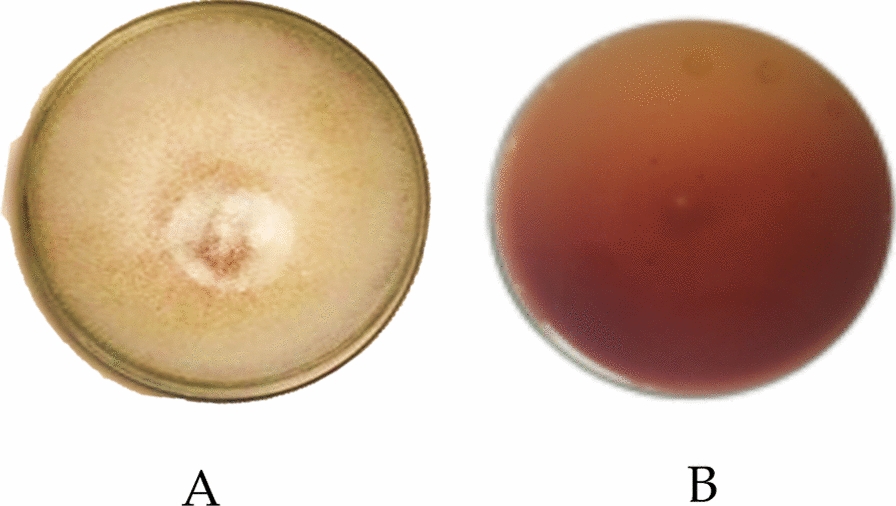
**A** Pure mycelium of *Ganoderma gibbosum,* **B** Red brownish zone of oxidoreductase laccase with guaiacol

Mycelium growth started on the 4th day following inoculation, as indicated by a sharp increase of laccase activity on the agar medium containing 0.02% guaiacol. The replicates (plates) were incubated (at 30 ℃ for 7 days), and the formation of (reddish-brown oxidation) zone on the agar plate indicated the ability of this species to release the laccase after oxidation reaction with guaiacol (Fig. 4 A, B). This culture medium helped in the preliminary assessment of laccase secretion from the species, and the oxidized zone of laccase (maroon brown) indicated the potential level of action. During laccase action with guaiacol, the formation of cyclohexa-3,5-diene-1,2-dione product changed the color of the agar medium.

### Optimization of Laccase activity and Dye decolorization

As an overall, statisitcally, the enzyme activity was non-significant compared to the used concentrations, while the discolaration rate showed highly signifincat results compared to the used concentrations with a p value of 4.67×10^-14^. The mean values of the enzymatic acitivites ranged from 43.93 ± 17.95 U/mL to 25.62 ± 14.85 U/mL. Regarding the discolaration rate it showed a range of values from 90.5 ± 3.55% to 61.25 ± 6.18% (Table 1.)

**Table 1:** Mean activities of laccasse enzyme and discolaration rate against the used different concentrations

Concentration (ppm)	Mean activity (U/mL) ± SD	Mean decolorization rate (%) ± SD
5	43.93^a^ ± 17.95	90.5^a^ ± 3.55
10	39.13^ab^ ± 17.19	85.42^ab^ ± 9.2
15	35.73^ab^ ± 14.76	81.08^b^ ± 8.32
20	31.6^ab^ ± 14.12	70.58^c^ ± 6.87
25	25.62^b^ ± 14.85	61.25^d^ ± 6.18

#### a. First batch (5 ppm)

**Fig. 5:**
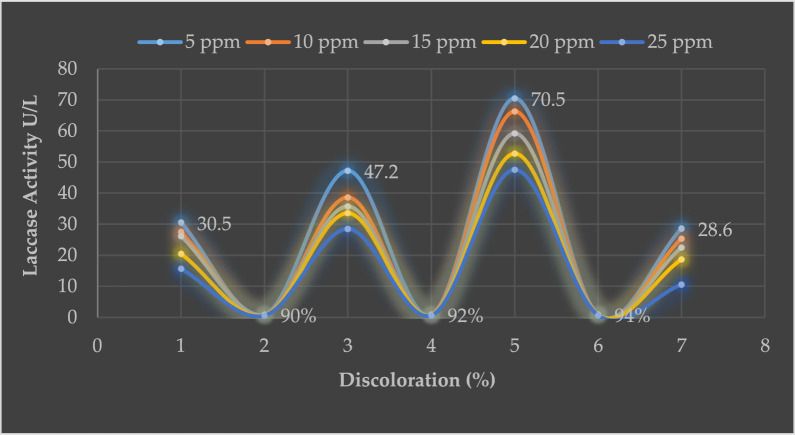
Laccase activity and rate of decolorization (%) of RBBR in the fungal fuel cell

Mycelium began to grow at the cathodic region after 4th day following inoculation, indicated by a sharp increase in laccase activity. The activity was 30.5 ± 0.6 U/L on day 4, reaching a plateau of 47.2 ± 0.2 U/L on day 6. Laccase-catalyzed RBBR started to decolorize the medium on day 6. The decolorization rate peaked on days 8 to 10, and laccase was 70.5 ± 0.4 U/L. As the culture aged, the laccase activity (28.6 ± 0.6 U/L) declined rapidly after 11 to 15 days. During the operation of FFC, the decolorization of the anolyte usually reached 92% in 4 to 6 days. Fresh RBBR solution was added periodically to restore the substrate concentration. In the early stage of the operation before day 7, FC decolorized the RBBR faster than the days after 11 to 15 (similar decolorized rate). The discoloration was 92% on 6 to 7 days and 94% from days 8 to 10. The rate declined 85% from day 10 to onward (Fig. 5).

This trend was paralleled by the changes in voltages over the same period. Mycelium at the cathode increased the decolorization of dye by several mechanisms. First, fungal mycelium functioned as an oxygen barrier for the anode chamber and kept the fully anaerobic conditions there. This favors maximum dye reduction. Secondly, laccase produced by the mycelium on cathode also increased the dye reduction rate. The fungal colony thus served as a continuous pollutant sink made possible by using PVA-H gel.

This gel allowed RBBR and its degradation intermediates to move freely between the electrodes and helped to prevent the anolyte acidification during prolonged treatment. Most studies indicated that using PEM (proton exchange membranes) in FCs accumulates cations on the anode side, but selectively favors the passage of proton (H^+^). Non-proton cations that try to migrate toward the cathode tend to be stopped by the membrane interface. Maximum days of treatment build-up maximum cations concentration near the membrane surface. In turn, a significant resistance for passage of H^+^ and flow of current lead to rapid acidification of the anolyte.

#### b. Second batch (10 ppm)

The second concentration of 10 ppm dye solution was decolorized by laccase action. The laccase activity was 27.5 ± 0.4 U/L in the cathodic region after the 4th day, sharply increased in laccase activity afterward as mycelium growth increased. The activity was 38.6 ± 0.6 U/L on day 6. Laccase-catalyzed oxidation of RBBR started on day 6. The decolorization rate caught the peak on days 8 to 10 days, and laccase was 66.5 ± 0.4 U/L, but a decline in laccase activity (25.4 ± 0.2 U/L) was observed as the culture grew old and exhausted rapidly after days 11. The decolorization of the anolyte reached 89% on days 4 to 6. In the early stage of the operation, before day 7, the FC decolorized the RBBR faster. The discoloration was 90% on 4 to 6 day and 92% from day 8 to 10. The rate declined by 70% from day 10 onwards (Fig. 5).

#### c. Third batch (15 ppm)

The results exhibited that the dye was highly decolorized when dye concentrations were low. The higher laccase amounts effectively removed the dye than the lower amount. This concluded that the increased amount of laccase gradually increased the color removal. The laccase activity 26.2 ± 0.5 U/L removed 86% dye from the 1.5 mL solution, while 35.6 ± 1 U/L removed 82% dye on the first 4 and 7 days, respectively. Similarly, 59.2 ± 0.2 U/L efficiently removed 89% dye on day 10, while 22.5 ± 0.7 U/L laccase efficiency declined to 68% on day 10 onward (Fig. 5).

#### d. Fourth batch (20 ppm)

Laccase 20.5 ± 0.5 U/L was efficient and sharply removed 70% of the dye concentration from the water in the first 4 days. This higher amount (33.5 ± 0.8 U/L) of laccase efficiently removed 75% of RBBR on day 7, whereas 77% on day 10 under action of 52.7 ± 0.3 U/L laccase treatment. This study concluded that the maximum quantity of laccase from *G. gibbosum* was efficient in removing dye. The efficiency of laccase in decolorizing the blue dye made the enzyme to be prospective for further industrial and biotechnological applications. The efficiency declined by 60% as the laccase activity decreased (18.7 ± 0.2 U/L) in 11 to 15 days (Fig. 5).

#### e. Fifth batch (25 ppm)

In this work, 25 ppm was the highest concentration used to evaluate the laccase action in discoloration. The laccase retained its decolorization activity against the dye after 9 days. The laccase gradually decreased in working when 15.7 ± 0.7 U/L removed 60% dye from the anodic chamber, while 65% on day 7, whereas 28.5 ± 0.7 U/L laccase was efficient in the removal of 65% on day 7 and 69% on day 10. As the dye concentration increased, the laccase efficiency decreased (10.6 ± 0.2 U/L) and removed 52% dye.

### Generation of electricity by fungal cathode

Mostly, the anodic reaction of a FC is catalyzed by microorganisms, where the reduction of O_2_ at the cathode was catalyzed by non-biological catalysts (platinum). Oxidative enzymes (market-based purified enzymes, i.e., laccase) are sometimes immobilized on the surface of the cathode during catalytic reaction. However, noble metals become corrosive, and enzyme catalysts are expensive and deteriorate in moist conditions during fuel cell operation. So, in this study, laccase-secreting *G. gibbosum* was applied onto the cathode to increase the power density of a dual fuel cell chamber at the cost of supplementation of nutrients and mediators for fungal growth and laccase action, respectively. *G. gibbosum* served as both a continuous source of fresh laccase and an aerobic dye-degrading organism.

**Fig. 6:**
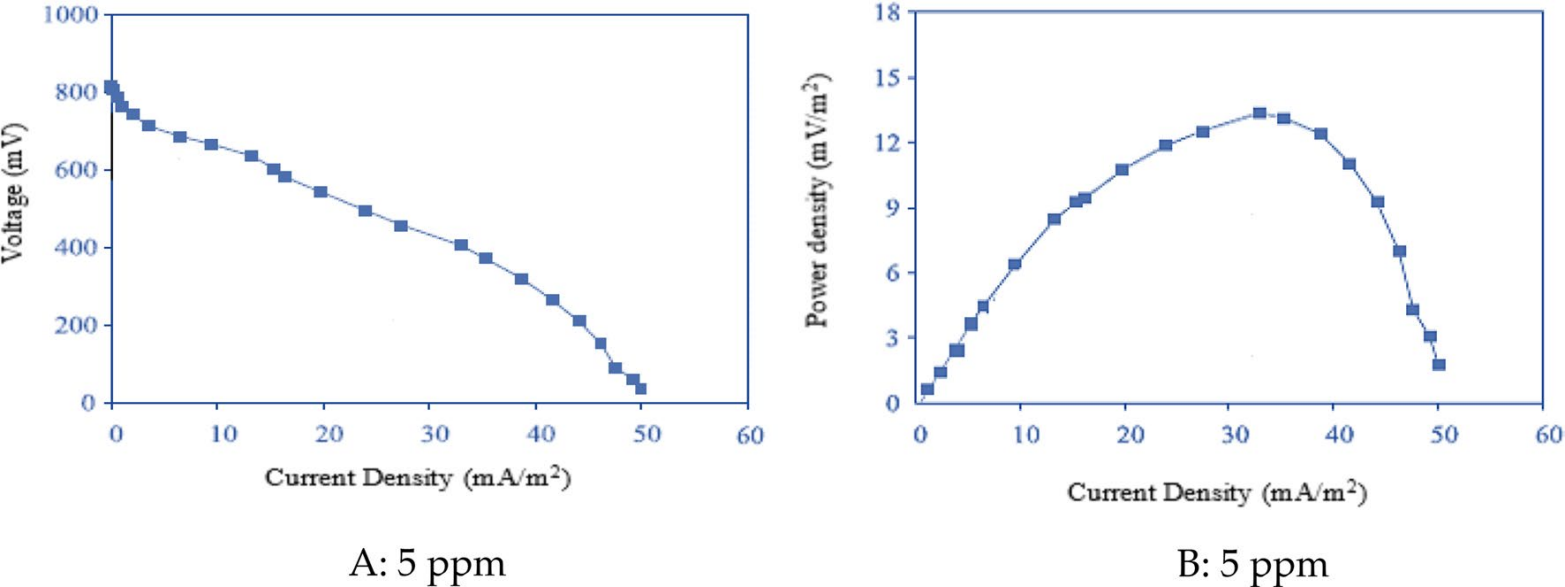
**A** Polarization and **B** Power density curves plotted for 5 ppm concentration for calculation of power generation after 10 days exhibited maximum laccase activity and discoloration of dye

This study stabilized the dual chamber for 15 days, and voltage was monitored continuously after dye addition. In the early stage, between days 5 to 10, reaching a maximum voltage of 810 mV on day 10. After the 10th day, the voltage of the cell declined slowly and eventually stopped on day 18. In the later stage, between days 18 and 20, the loss rate increased the potential of the FC because of excessive fungal mycelium growth on the cathode and membrane. The maximum thickness of the mycelium layer minimized oxygen availability to the cathode. The distance became maximum amongst active laccase-producing growing tips of mycelium, and the electrode was responsible for reducing the laccase activity (U/L) on the electrode surface. The internal resistance of the FCs was examined herein by using the polarization slope method and power density peak method [14]. Figure 6 (A & B) was plotted for polarization and power density curves recorded for 15 days continuously during operation. The maximum power density at current densities of 35 mA/m^2^ was 14.18 mW/m^2^. The reduction of more than 100 omega in the internal resistance can be attributed to a decrease in the activation loss of laccase activity. Thickened fungal mycelium during prolonged operation did not increase internal resistance because it was planted on the outer surface of the cathode and continuously grew away from the cathode into the air.

## Discussion

A modern society without using chemicals in pulp, leather, pharmaceutical, and paper industries is impossible. However, consuming chemicals contaminate the environment and cause harmful effects [16]. Chemical and physical methods include electrochemical methods for wastewater decolorization [17]. These methods are relatively expensive, have low removal efficiencies, produce toxic intermediates, and exhibit high specificity for dyes [18].

Biological processes degrade the dyes of wastewater through decolorization by fungal strains [19]. These processes are slow; however, their efficiency is satisfactory. Fungal fuel cells are an emerging technique that effectively treat the wastewater and simultaneously generate electricity. The performance of a fuel cell is estimated in terms of pollutant removal and electricity production. Wastewater pollution from numerous sources can be treated by FFCs, where organisms decompose organic compounds and convert this chemical energy into electrical energy [2]. The advantages of this method are a low concentration of reagents needed under mild conditions and degrade a wide range of substrates.

Enzymatic decolorization is now used for the decolorization of effluents [19]. Extracellular ligninolytic oxidative enzymes, e.g., valuable extracellular oxidoreductases (laccases), help the fungi to degrade dyes [20]. Multiple enzyme systems successfully break the diverse organic pollutants by oxidation or degradation into smaller intermediates. Oxidoreductase is renowned for degrading numerous organic pollutants [21]. Reactive diffusible redox mediators (RMs) based on oxidoreductase dramatically increased the reaction rate of a broad range of substrates degraded by these enzymes [22]. Laccase has high catalytic efficiency, low substrate specificity, and requires minimum reaction time. The parameters for the reaction are simple and do not produce harmful byproducts [23]. The high catalytic potential of this enzyme can treat wastewater of industries with biotransformation of the dyes [24].

RBBR an anthraquinone, and the second most crucial textile dyes class belongs to hazardous and resistive pollutants. The following equation indicated how the dye (RBBR) degraded by laccase into low biodegradable compounds, and water was released as a byproduct during the laccase mechanism of action. Overall, laccase exhibited better performance in terms of textile dye removal. The reactiveness of laccase secreted by *G. gibbosum* was maximum due to the presence of media supplements.
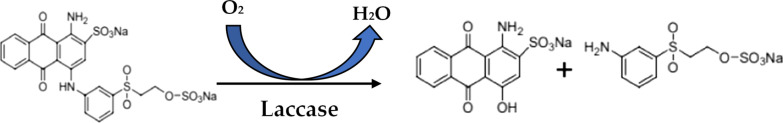


During RBBR degradation, laccase performed redox, hydroxylation, and deamination reactions. In the oxidation reaction of enzyme–substrate complex, molecular O_2_ is required from the atmosphere, and reduction of O_2_ into water takes place. Instead, H_2_O_2_ is needed by other oxidative enzymes. So, this action mechanism is eco-friendly, because oxidation and intramolecular electron transfer to proton allow the O_2_ reduction into H_2_O. The use of oxygen in laccase reaction has sparked the interest at the industrial level, and O_2_ can be used as a primary oxidant, possibly controlling the injection or decrease of O_2_ pressure during the enzymatic reaction by the laccase of this species. The formation of low-molecular-weight products after laccase-catalyzed reaction processed low toxicity levels. Therefore, many researchers studied the laccases, because of dye decolorization efficiency [25–29]. This study used laccase extracted from *G. gibbosum* to successfully decolorize RBBR via fungal fuel cells in a pollution-free environment. Table 2 indicated the literature where laccase from different wood-rotting fungi was used in dye decolorization.

**Table 2:** Literature indicated % discoloration of dyes

Sr No	Species Name	Laccase	Dye name	% Discoloration	References
1	*Pleurotus ostreatus*	HAUCC 162	Synthetic dyes	-	[30]
2	*Pleurotus ostreatus*	LACC6, LACC9, LACC10	Malachite Green (MG), Remazol Bright Blue R (RBBR), Bromophenol Blue (BB), and Methyl Orange (MO)	LACC6 (MG-91.5%, RBBR-84.9%, BB-79.1%, MO-73.1%), while LACC10 (71.1–54.8%), LACC9 (7.1–67.9%)	[31]
3	*Coprinus comatus*	Lac4, Lac3	Bright Blue Reactive X-BR (BB X-BR), Bright Blue Reactive K-GR (BB K-GR), Bright Blue Reactive K-3R (BB K-3R), Orange Reactive 1 (RO), Reactive Red X-3B (RR X-3B), Congo Red (CR), Dark Blue Reactive KR (DBR KR), Coomassie G-250 (C), Victoria Blue, Methyl Violet	Lac3 (~ 67% and 90%) removed RBBR, BB K-GR, DBR KR, and (~ 33% and 48%) BB X-BR, CR, C, VM, and VB	[32]
4	*Trametes* sp. 48,424	rLAC48424- 1	“MO, MG, BB and Violet Crystal (CV)”	MG (97%) BB (~ 90%), and CV (68%)	[33]
5	*Trametes trogii*	lcc1	Alizarin Red (AR), Carmoisine (CM), Cochineal Red (CCR), Sunset Yellow (SY), Patent Blue (PB), and Indigo Blue (BI)	AR (~ 23%), CM (87%), CCR (75%), SY (67%), PB (81%)	[34]
6	*Polyporus* sp.	S133 laccase	RBBR	90%	[35]
7	*Trametes pubescens*	Tplac	Congo Red (CO),		[36]
8	*Oudemansiella canarii*	Lacc	RBBR	100 mg/L	[37]
9	*Ganoderma* sp. KU-Alk4	Lacc	Commercial aromatic dyes	100%	[38]
10	*Ganoderma lucidum*	Lacc	CO	80%	[39]
11	*Ganoderma lucidum*	Lacc	Textile dye wastewater	81.4%	[40]

The reduction rate on abiotic cathodes heavily depends on the dye molecular structure [41]. As many dyes are laccase substrates, and laccases use as a cathode catalyst effectively increased the dye discoloration range. White-rotters are effective dye degraders, and laccase a dye-degrading enzyme was produced in this study. Most dyes and their degradation intermediates can be absorbed and assimilated in the presence of living fungal cells and their complete metabolic network.

Intra- and extracellular enzymes are helpful in fungal metabolic activities and the degradation of different dyes from textile wastewater. In bioanode–biocathode FFCs, the dye wastewater acts as anolyte and catholyte. The maximum decolorization was observed in the first 12 h, because a higher substrate consumption at the cathode and anode occurs during the initial hours. The maximal cell potential is recorded to be 706 mV in a fungal FC, with power densities of 276.9 mWm^−2^. The reactor was also tested for the biodegradation of RR 195 dye from wastewater, bioelectricity production, and 95% removal efficiency [42]. Dye decolorization was carried out in a fungus yeast-mediated single-chambered FC. The maximum power density was recorded (34.99 mW m^−2^) on the 21st day. The best response to dye decolorization was observed in MB9 (96%) followed by RBBR (90–95%), AR1 (38%), and OG (76%) [43]. The decolorization of wastewater containing different dyes occurred via the movement of e^−^ from anode to cathode by an external circuit. Azo dyes in the catholyte act as e^−^acceptors and dyes are decolorized via reductive cathode reactions. The dye-reducing reactions progress better under anaerobic conditions, where O_2_ competes for e^−^ from the cathode, and the reaction rate heavily depends on the pH of the catholyte [44].

The power density and cell voltage increased, when the dye concentration was minimal, while the ‘V’ and ‘P” as the dye concentration increased in this study. Increased current reduction in many laccase-modified carbon electrodes may be due to two mechanisms: (1) direct e^− ^transfer from the electrode to the T1 copper ion of laccase; (2) mediated e- transfer between the electrode and laccase [45]. In the latter mechanism, free radicals were generated by laccase, catalyzed 1e^−^oxidation of phenolic substrates frequently act as mediators. When the dye was omitted from the anolyte, a mediated mechanism of dye or its oxidized products as mediators was more likely to be involved in the fungal cathodes.

## Conclusion

In this study, *G. gibbosum* was identified by ITS marker, and phylogenetic tree facilitated in species identification. The laccase secreted by this species successfully decolorized the Remazol Brilliant Blue R (RBBR) dye along with electricity generation via fungal fuel cell. This species can be used in Fuel Cell Technology (FCT) in eco-friendly manner at industrial levels. The overall conclusion of this work suggested that laccase is valuable for industrial applications and plays a significant role in dye decoloration along with current production. 

## Data Availability

The data set generated and analyzed during the current study is available from the corresponding author on personal request. The consensus was deposited to GenBank under accession number OM350473, OM350446.
